# CD4 Cell Counts at HIV Diagnosis among HIV Outpatient Study Participants, 2000–2009

**DOI:** 10.1155/2012/869841

**Published:** 2011-09-20

**Authors:** Kate Buchacz, Carl Armon, Frank J. Palella, Rose K. Baker, Ellen Tedaldi, Marcus D. Durham, John T. Brooks

**Affiliations:** ^1^Centers for Disease Control and Prevention, Atlanta, GA 30333, USA; ^2^Cerner Corporation, Vienna, VA, USA; ^3^Northwestern University, Chicago, IL, USA; ^4^Temple University, Philadelphia, PA, USA

## Abstract

*Background*. It is unclear if CD4 cell counts at HIV diagnosis have improved over a 10-year period of expanded HIV testing in the USA. *Methods*. We studied HOPS participants diagnosed with HIV infection ≤6 months prior to entry into care during 2000–2009. We assessed the correlates of CD4 count <200 cells/mm^3^ at HIV diagnosis (late HIV diagnosis) by logistic regression. *Results*. Of 1,203 eligible patients, 936 (78%) had a CD4 count within 3 months after HIV diagnosis. Median CD4 count at HIV diagnosis was 299 cells/mm^3^ and did not significantly improve over time (*P* = 0.13). Comparing periods 2000-2001 versus 2008-2009, respectively, 39% and 35% of patients had a late HIV diagnosis (*P* = 0.34). Independent correlates of late HIV diagnosis were having an HIV risk other than being MSM, age ≥35 years at diagnosis, and being of nonwhite race/ethnicity. *Conclusions*. There is need for routine universal HIV testing to reduce the frequency of late HIV diagnosis and increase opportunity for patient- and potentially population-level benefits associated with early antiretroviral treatment.

## 1. Introduction

Recent HIV surveillance data suggest that approximately 33% of HIV-infected persons in the United States present for HIV testing late and have AIDS (CD4+ cell count <200 cells/mL or an AIDS-defining illness) within one year after HIV diagnosis [1, 2]. Patients are less likely to experience the full benefits of highly active combination antiretroviral (cART) therapy if they enter HIV care and initiate treatment at a CD4 count <350 cells/mm^3^ [3, 4]; the clinical cost is even more profound when the CD4 count is <200 cells/mm^3^ or the patient has already developed clinical AIDS [5–8]. In addition, persons who remain unaware of their HIV-positive status (estimated 21% to 25% of infected persons in the USA in recent years) [9, 10] may not only miss the benefits of earlier cART treatment, but are also more likely to remain chronically viremic and are thereby more likely to transmit HIV to their sexual and needle-sharing partners [9]. 

The CDC has been promoting strategies to encourage more widespread HIV screening to diagnose infected persons earlier in the course of their illness, including by releasing in 2006 the guidelines for implementing routine universal opt-out testing in healthcare settings [11]. Yet, the latest HIV surveillance data [1, 2] and epidemiologic studies in multiple US populations indicate that the proportion of persons who are diagnosed late in the course of HIV infection [2, 12, 13] or present late for HIV care [14, 15] remains unacceptably high. Stable or worsening trends in the proportion of patients HIV diagnosed with low CD4 counts have also been observed internationally [16–18]. However, encouraging trends have been seen in select US jurisdictions which have dramatically expanded their HIV testing programs [19, 20]. With the recent shift in antiretroviral treatment guidelines toward earlier therapy initiation to benefit patients' health [21] and the growing interest in HIV “test and treat” and “test and link to care-plus” strategies to limit the spread of the US epidemic [22], it is important to understand the burden of late HIV diagnosis among contemporary US patients as it affects the likelihood of success of these interventions.

We studied participants in the HIV Outpatient Study (HOPS) who were recently diagnosed with HIV infection to examine the trends in median CD4 count at diagnosis and the proportion of patients diagnosed with CD4 count <200 cells/mm^3^ (also termed “late HIV diagnosis”) during 2000–2009.

## 2. Methods

### 2.1. The HIV Outpatient Study (HOPS)

The HOPS is an ongoing, open prospective observational cohort study that has continuously recruited and followed HIV-infected patients since 1993. Since the HOPS' inception, the study sites have included 10 clinics (6 university, 2 public, 2 private) in eight US cities and provided care for about 3,000 HIV-infected patients per year. Over 9,600 HOPS patients have been seen during more than 390,000 clinical encounters. The study protocol is approved and renewed annually by each participating institution's ethical review board. All study participants provide written, informed consent.

HOPS clinicians have extensive experience treating HIV-infected patients. Information is abstracted from outpatient charts at each visit, entered electronically by trained staff, compiled centrally, and reviewed and edited before being analyzed. Abstracted information includes demographic characteristics and risk factors for HIV infection; diagnoses; symptoms; prescribed medications, including dose and duration; laboratory values, including CD4 counts and plasma HIV viral loads; causes of mortality and hospitalizations.

### 2.2. Study Population

 We analyzed data from HOPS participants using the dataset updated as of March 30, 2011. The 10 clinics included in the analyses were located in Tampa, FL (2 sites); Washington, DC; Denver, CO (2 sites); Chicago, IL (2 sites); Stony Brook, NY; Oakland, CA; Walnut Creek, CA, and Philadelphia, PA. We limited analyses to patients who had been HIV-diagnosed during 2000–2009, within ≤6 months before entry into care at a HOPS site (i.e., had a recent HIV diagnosis), had complete records of antiretroviral use (if any), and had a CD4 count measured at the time of HIV diagnosis (up to 3 months after HIV diagnosis date) and before having received any antiretroviral treatment. Thus we focused on subset of patients who entered HOPS care after a recent (i.e., within the past 6 months) diagnosis of HIV infection, for whom we had more complete data on CD4 count at HIV diagnosis.

We defined medical coverage/insurance (insurance) as private for the following payer categories: health maintenance organization, preferred provider organization, point of service plans, “self-pay/fee-for-service,” and “other private insurance.” We defined the following payer categories as public insurance: Medicaid, Medicare, Ryan White/AIDS Drug Assistance Program, and “public, state funded.” We defined the categories other, unknown, and “clinical study” as other or unknown insurance.

We classified the following HOPS sites, which serve diverse patient populations, including indigent patients, as public facilities: State University of New York (SUNY), Stony Brook, NY; Temple University School of Medicine, Philadelphia, PA; University of Illinois at Chicago, Chicago, IL. The remaining institutions listed in the Acknowledgments were classified as private facilities.

### 2.3. Analysis Methods

We used chi-square or Fisher's Exact test to analyze categorical variables and the Wilcoxon rank sum test to compare distributions of continuous variables. We examined median CD4 counts and the proportion of patients having a CD4 count <200 cells/mm^3^ at HIV diagnosis during the entire period 2000–2009 and by five two-year periods within this time frame: 2000-2001, 2002-2003, 2004-2005, 2006-2007, and 2008-2009. We assessed temporal trends in the distribution of CD4 counts at HIV diagnosis using the Jonckheere-Terpstra nonparametric test and assessed trends in the proportion of persons diagnosed with CD4 count <200 cells/mm^3^ by calendar period using the Cochran-Armitage test. Factors associated with having a CD4 count <200 cells/mm^3^ at HIV diagnosis (also termed “late HIV diagnosis” henceforth) were examined using multiple logistic regression. Factors considered in the logistic models included patient's age, gender, race/ethnicity, HIV risk, and patient's insurance (private versus public or none). We chose to evaluate patient's insurance rather than the type of HOPS site (private versus public facility) in the primary multivariable model because these two variables were correlated and because we hypothesized that patient's insurance would be more closely tied to behaviors and circumstances associated with late HIV testing; we evaluated type of HOPS facility, in place of patient's insurance, in an alternate multivariable model. 

We also performed additional analyses of trends in CD4 count at diagnosis after transforming data on the square-root scale. The data describing the location of initial HIV diagnosis were collected systematically since mid-2005 and were analyzed only for patients diagnosed in 2006–2009.

Finally, we evaluated the percentage of persons who developed AIDS (defined as CD4 count of <200 cells/mm^3^ or CD4+  T-lymphocyte percentage of total lymphocytes of <14 or documentation of an AIDS-defining condition) within 12 months of HIV diagnosis [1].

## 3. Results

Of 3,670 new HOPS participants seen at 10 HIV clinics during 2000–2009, 1,223 (33%) had a recent HIV diagnosis (≤6 months before entry into care at a HOPS site). Compared with the 2,447 patients who were diagnosed with HIV infection >6 months prior to entry into care at a HOPS site (and may have been in care elsewhere), recently diagnosed patients were significantly (*P* < 0.05) more likely to be ARV naïve (86% versus 16%), younger (median age of 38 versus 40 years), to have heterosexual activity as their sole risk for HIV infection (37% versus 25%), to be privately insured (61% versus 51%), less likely to be male (75% versus 80%), less likely to be of white race (40% versus 47%), and less likely to have injection drug use (IDU) activity as their sole risk for HIV infection (6% versus 11%). 

Of the 1,223 patients with recent diagnoses, 936 (77%) had a CD4 count documented within 3 months following HIV diagnosis while still antiretroviral naïve (a median of 15 days, IQR: 4–34 days after diagnosis). Of these 936 patients (median age = 38, range: 17–77 years), 77% were male, 43% were white, 39% were black, 54% were men who had sex with men (MSM), 36% had heterosexual HIV risk, and 5% were injection drug users (Table 1). The 23% of patients whom we excluded because they did not have a CD4 count documented in the relevant timeframe were significantly more likely to have had a history of IDU (9% versus 5%), to have public insurance (41% versus 29%), and to have been antiretroviral-experienced (23% versus 7%) at entry into care at a HOPS site.

### 3.1. Trends in CD4 Counts at HIV Diagnosis

The overall median CD4 count at HIV diagnosis was 299 cells/mm^3^ (mean = 339, IQR 100–498); among patients diagnosed in 2000-2001 and 2008-2009 the median CD4 counts were 284 cells/mm^3^ and 314 cells/mm^3^, respectively, (*P *value for trend across the five two-year time periods = 0.13) (Table 2). We also found no linear trend in CD4 counts at diagnosis by calendar period after data were transformed on the square-root scale to normalize the distributions and were analyzed by generalized linear models (data not shown). Although not statistically significant, there was a trend toward improvement in CD4 cell counts among patients who had heterosexual contact as a risk factor for HIV infection and those seen in public HOPS clinics (Table 2).

### 3.2. Low CD4 Counts at HIV Diagnosis

**Figure 1:**
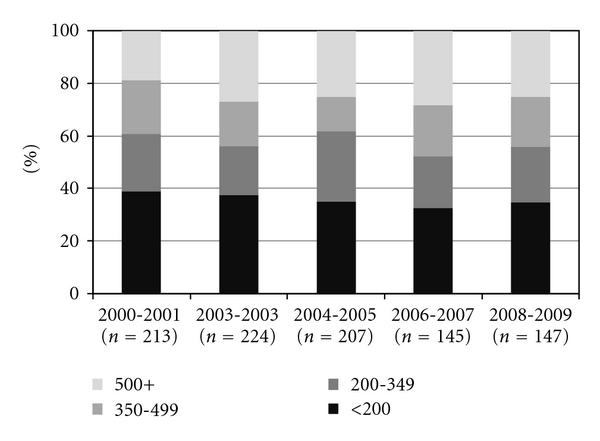
CD4 cell count (cells/mm^3^) by period of HIV diagnosis, the HIV Outpatient Study 2000–2009 (*N* = 936).

Among the 936 recently diagnosed patients who had CD4 cell count data available, 337 (36%) were diagnosed with a CD4 count <200 cells/mm^3^ (i.e., had a late HIV diagnosis), 39% of patients diagnosed in 2000-2001 and 35% of patients diagnosed in 2008-2009 (Cochran-Armitage test for trend across the five two-year periods, *P* = 0.21) (Figure 1). During 2000–2009, late HIV diagnoses were significantly (*P* < 0.05) more common among black (42%) and Hispanic (46%) patients compared with white patients (28%), among patients with public (42%) versus private insurance (34%), and among patients entering care at public versus private HOPS clinics (45% versus 30%, resp.). The frequency of late HIV diagnoses was significantly lower among MSM (27%) compared with all other risk groups (47%).

Five hundred forty-one (58%) patients were HIV-diagnosed with a CD4 count <350 cells/mm^3^, 61% of patients diagnosed in 2000-2001 and 56% of patients diagnosed in 2008-2009 (Cochran-Armitage test for trend across the five two-year time periods, *P* = 0.29). The percentages of HIV diagnoses made among persons with a CD4 count <350 cells/mm^3^ were also significantly higher for blacks (63%) and Hispanics (65%) compared with whites (50%), for patients with public (63%) versus private (55%) insurance, and for patients entering care at public versus private HOPS clinics (66% versus 53%, resp.). The frequency of HIV diagnoses made with an initial CD4 count <350 cells/mm^3^ was lower among MSM (50%) compared with all other risk groups (67%). Only about 25% of patients were HIV-diagnosed with CD4 cell counts ≥500 cells/mm^3^, and could potentially benefit from early antiretroviral treatment initiation per the latest US guidelines [21].

Univariate analyses of factors associated with an HIV diagnosis at a CD4 count <200 cells/mm^3^ did not reveal substantive differences in associated predictors across the five two-year analysis periods (data not shown). In univariate logistic regression analyses for all patients (*n* = 936), factors associated with late HIV diagnosis included white race/ethnicity (odds ratio OR = 0.53, 95% confidence interval CI: 0.40–0.70); age <35 years old (OR = 0.45, 95% CI: 0.32–0.64 versus age 35–42 years old); having as a risk for HIV infection not being MSM, or in other words, having high-risk heterosexual, IDU, or another risk (e.g., hemophilia, blood transfusion or occupational exposure) (OR = 2.49, 95% CI: 1.90–3.28 versus MSM), and having public insurance at entry into care at a HOPS site (OR = 1.43, 95% CI: 1.07–1.93 versus private insurance). 

In multivariable logistic regression analyses, independent correlates of HIV diagnosis with CD4 count <200 cells/mm^3^ were having as a risk for HIV infection not being MSM (OR = 1.99, 95% CI 1.45–2.72), age ≥35 years at diagnosis (OR = 2.14, 95% CI 1.59–2.87), and being of nonwhite race (OR = 1.45, 95% CI 1.05-2.01). The association between late HIV diagnosis and having public insurance that was observed in the univariate analyses did not persist in the adjusted analyses. In an alternate multivariable model, which included type of HOPS site (public versus private facility) instead of patient's insurance, the type of HOPS site was also not associated with late HIV diagnosis after controlling for patient's age, race/ethnicity, and HIV risk group.

### 3.3. AIDS within 1 Year of HIV Diagnosis

Because documentation of AIDS opportunistic illnesses may be incomplete at the time of initial HIV diagnosis, we evaluated the percentage of persons who developed AIDS (by immunologic or clinical criteria) ≤12 months of HIV diagnosis. Across the five two-year periods, respectively, that percentage ranged from 50.2% for patients diagnosed in 2000-2001 to 50.3% for patients diagnosed in 2008-2009 (test for trend *P* = 0.66).

### 3.4. Circumstances of the Initial HIV Diagnosis

Data on the venue of initial HIV diagnosis were available for 246 of 292 patients diagnosed in 2006–2009. The predominant situations in which HIV diagnoses were made included screening at a routine provider visit (*n* = 75, 30%), testing during inpatient hospitalization (*n* = 43, 17%), testing at a symptom-driven visit (*n* = 29, 12%), and testing at a sexually transmitted disease clinic (*n* = 25, 10%); the circumstances surrounding initial HIV diagnosis were recorded as “unknown” for 29 (12%) of participants.

## 4. Discussion

Among HOPS patients recently diagnosed with HIV infection, we found no statistically significant improvement in the median CD4 count at diagnosis during 2000–2009. Overall, 36% of patients were diagnosed with a CD4 count <200 cells/mm^3^ and 58% with a CD4 count <350 cells/mm^3^. Persons whose risk for HIV infection was other than being MSM, persons aged ≥35 years, and persons of nonwhite race/ethnicity were more likely to be diagnosed with a CD4 count <200 cells/mm^3^ and thus more likely to have missed an opportunity for timely access to HIV care and initiation of ARV therapy; the correlates of HIV diagnosis with CD4 <350 cells/mm^3^ were largely similar. Our finding that MSM were less likely to be diagnosed with advanced HIV infection than some other risk groups (e.g., IDUs) is consistent with the findings from US HIV surveillance [1, 2] and data from other HIV cohorts reporting on late HIV diagnosis [23] and presentation for care [15]. The association of younger age (<35 years) with a lower likelihood of late HIV diagnosis may be partially explained by the fact that younger persons *de facto* have had less lifetime opportunity, if they became HIV-infected, to progress to CD4 <200 cells/mm^3^; older age has been associated with late HIV diagnosis previously [2, 15].

In a recent study by Althoff and colleagues of 44,491 HIV-infected patients enrolled in the North American-AIDS Cohort Collaboration on Research and Design (NA-ACCORD), the median CD4 count at first presentation to HIV care increased from 256 cells/mm^3^ (interquartile range, 96–455 cells/mm^3^) to 317 cells/mm^3^ (interquartile range, 135–517 cells/mm^3^) from 1997 to 2007 (*P* < 0.01). The median CD4 count at HIV diagnosis for HOPS patients whom we studied (who all entered care within 6 months of diagnosis, per inclusion criteria) was remarkably similar to median CD4 count at first presentation to HIV care among NA-ACCORD participants. Our findings of a high (approximately 34%) prevalence of late HIV diagnosis during the period 2004–2007 correspond well with the estimate from national HIV surveillance (34%) for 2005–2007, and the national surveillance system also detected no marked increases in CD4 counts at diagnosis over time [1]. The reason why the median CD4 counts in the HOPS and NA-ACCORD population were higher as compared with those in HIV surveillance may be related to several factors. First, compared with the nationwide HIV epidemic, the HOPS is enriched in whites and MSM, populations who tend to be diagnosed at higher CD4 cell counts. Second, HIV testing capacity and therefore the opportunity for timely diagnosis vary by jurisdiction. HOPS clinics are located in major urban centers with large HIV epidemics where testing opportunities might be greater leading to a greater likelihood of indentifying infected persons earlier in the course of their disease. Third, the CD4 count findings from national HIV surveillance are derived from 37 states, some of only report CD4 counts <200 cells/mm^3^ for HIV-infected persons, a practice that would tend to bias the median CD4 cell count downwards [2].

Our results should be interpreted in light of some additional important caveats. First, we restricted our analyses to patients diagnosed with HIV within 6 months prior to entry into care at a HOPS site. We used this criterion because HOPS patients who initiated HIV care earlier elsewhere often lack documentation of their initial CD4 count value. The timeframe of ≤6 months between HIV diagnosis and entry into care at a HOPS site, however, appeared to capture well the patients who newly entered care (only 7% of these patients had any previous ARV exposure). Although we have thus excluded a subset of patients who delay entry into HIV care (who may also be more likely to be diagnosed late in HIV infection) a recent meta-analysis suggests that approximately 72% of patients enter care within 4 months of HIV diagnosis [24]. Second, we studied a self-selected group of recently diagnosed patients who, by definition, entered HIV care and were also willing to consent to enrollment in the HOPS; these patients could potentially differ from patients not entering care [25, 26]. Available resources constrain some HOPS sites from enrolling the entire clinic population, and although attempts are made to balance the representativeness of the enrollees there is some opportunity for convenience sampling bias. Additionally, the HOPS does not enroll patients who present to a HOPS clinic for care but die prior to the opportunity to consent for the study. Both of these effects are greater at public facilities compared with private facilities. Consequently, there might be a tendency for the HOPS to enroll healthier patients, and, therefore, our findings would not represent the entire clinic population served by HOPS sites or be generalizable to all patients in cities where HOPS sites are located. Third, the 23% of patients with recent HIV diagnoses who were excluded due to missing CD4 count data were more likely to be IDU and publically-insured, subgroups that tended to have lower CD4 counts at diagnosis. Therefore our finding that approximately one-third of patients were diagnosed with HIV infection at a CD4 count <200 cells/mm^3^ might be an underestimate due to this information bias. Finally, because of small sample sizes in some sociodemographic subgroups (e.g., Hispanics, high-risk heterosexuals, persons with a history of IDU) we could not accurately estimate median CD4 counts and proportions of patients with CD4 counts <200 at diagnosis by calendar period for these subgroups; furthermore, estimates for some groups (e.g., women) could be subject to considerable random variability over time.

The CDC recommendations for universal opt-out HIV testing were published in September 2006; in light of inherent reporting delays with HIV surveillance data and data incompleteness, the evidence as to whether these guidelines have led to earlier HIV diagnosis (i.e., at higher CD4 counts) nationwide is not likely to be apparent for some time. Nonetheless, encouraging trends have been seen in jurisdictions that have dramatically expanded HIV testing [19, 20]. In our study, we did not detect a statistically significant trend to higher CD4 cell counts at HIV diagnosis overall, although the pattern of change was in the direction of increasing CD4 cell counts, particularly for patients with heterosexual exposure as a risk factor for HIV infection and those presenting to public facilities. 

In conclusion we have found that even among HOPS participants who successfully entered care, over one-third were diagnosed with CD4 counts <200 cells/mm^3^ and over one-half were diagnosed with CD4 count <350 cells/mm^3^, the threshold at which initiating cART is unequivocally recommended (up to CD4 count of 500 cells/mm^3^, above which level it is to be considered) [21]. These findings should raise concern. Prompt HIV diagnosis, entry into care, and timely initiation of cART are critical for reducing the risk of both opportunistic and nonopportunistic disease, prolonging survival, and reducing onward HIV transmission. Our findings also suggest that expanding testing and reducing late HIV diagnosis need to be a priority, if the programs related to improving linkage to care and earlier antiretroviral treatment initiation [22] are to reach patients and potentially alter the trajectory of the US HIV epidemic [27]. An estimated 21 to 25% of HIV-infected persons in the USA remain undiagnosed [9, 10], with the prevalence of unrecognized HIV infection approaching 50% among some urban MSM [28]. Many untested persons either perceive themselves to be at low risk of HIV infection or are fearful of learning their HIV status [28]. Such persons are more likely to have a late stage or illness-triggered diagnosis. Our findings reinforce the need to establish universal routine HIV testing as standard of care for all adolescents and adults seen in private and public care settings, regardless of patient-reported HIV risk [11]. It is only under such circumstances that late-stage or illness-triggered HIV diagnoses will be reduced and that sociodemographic disparities in stage of HIV disease at diagnosis be eliminated.

##  Disclaimer

The findings and conclusions in this report are those of the authors and do not necessarily represent the views of the Centers for Disease Control and Prevention.

##  Conflict of Interests

None of the authors have conflict of interests.

## Figures and Tables

**Table 1:** Characteristics of patients diagnosed with HIV infection within 6 months before study entry with available CD4 count data* by study period during which HIV diagnosis occurred, the HIV Outpatient Study, 2000–2009.

	2000-2001 (*n* = 213)	2002-2003 (*n* = 224)	2004-2005 (*n* = 207)	2006-2007 (*n* = 145)	2008-2009 (*n* = 147)	*P* value for trend^†^	All years (*n* = 936)
*Males*, %	75	71	81	81	76	0.21	77
*Age, median years (IQR)*	37 (30–45)	38 (30–45)	39 (31–46)	36 (28–46)	36 (28–46)	0.50	38 (30–46)
*Race/ethnicity*, %							
Non-Hispanic white	44	39	47	48	36	0.67	43
Non-Hispanic black	37	44	36	34	44	0.83	39
Hispanic	15	13	14	14	18	0.59	15
Other or unknown	4	3	3	4	3	0.65	3
*HIV infection risk group*, %							
Men who have sex with men (MSM)	50	48	57	69	49	0.08	54
High-risk heterosexual	39	41	32	21	44	0.26	36
Injection drug use (IDU, including MSM IDU)	6	6	6	3	1	0.02	5
Other or unknown	5	6	5	6	7	0.54	6
*Medical insurer*, %							
Privately insured	57	67	67	70	58	0.51	64
Publically insured	34	29	29	24	33	0.37	30
Other or unknown	9	4	3	6	10	0.70	6
*Type of facility*, %							
Public	28	46	42	28	49	0.03	39
Private	72	54	58	72	51	0.03	61

*For inclusion in this analysis, patients must have had a CD4 cell count measured within 1 month prior to or up to 3 months after the date of HIV diagnosis while remaining antiretroviral-naïve.

^†^By Cochran-Armitage test for proportions (%), and by Jonckheere-Terpstra nonparametric test for continuous variables.

**Table 2:** Median CD4 cell count (cells/mm^3^) at HIV diagnosis* by demographic characteristics and study period during which HIV diagnosis occurred, the HIV Outpatient Study, 2000–2009. Table presents only strata including at least 100 patients in the study.

	2000-2001 (*n* = 213)	2002-2003 (*n* = 224)	2004-2005 (*n* = 207)	2006-2007 (*n* = 145)	2008-2009 (*n* = 147)	*P* value for trend^†^	All years (*n* = 936)
*Overall, median (interquartile range)*	284 (99–438)	298 (71–523)	288 (110–501)	320 (139–517)	314 (90–502)	0.13	299 (100–498)
*Overall, mean (95% confidence interval)*	312 (277–347)	346 (306–386)	341 (302–380)	354 (312–396)	353 (303–402)	—	339 (321–358)
*By gender*							
Male (*n* = 716)	272	320	293	355	305	0.34	304
Female (*n* = 220)	287	258	283	222	374	0.22	286
*By race/ethnicity*							
Non-Hispanic white (*n* = 400)	362	333	316	383	352	0.69	348
Non-Hispanic black (*n* = 365)	243	272	282	264	276	0.60	271
*By HIV infection risk group*							
MSM (*n* = 505)	351	375	308	386	336	0.80	351
Heterosexual (*n* = 372)	209	247	229	288	293	0.09	247
*By medical insurer*							
Privately insured (*n* = 596)	284	336	301	343	328	0.38	310
Publicly insured (*n* = 283)	230	267	234	213	294	0.27	260
*By type of facility*							
Public (*n* = 361)	208	225	277	212	276	0.10	247
Private (*n* = 575)	331	337	310	343	352	0.32	330

*For inclusion in this analysis, patients must have had a CD4 cell count measured within 1 month prior to or up to 3 months after the date of HIV diagnosis while remaining antiretroviral-naïve.

^†^By Jonckheere-Terpstra nonparametric test for continuous variables.

^‡^Differences in CD4 counts by race/ethnicity, HIV infection risk group, insurance, and type of facility were all statistically significant (nonparametric Wilcoxon rank sum test *P* < 0.05).
